# Neuropeptide Y modulates the electrical activity of subfornical organ neurons

**DOI:** 10.1016/j.crneur.2025.100149

**Published:** 2025-04-17

**Authors:** Lauren Shute, Mark Fry

**Affiliations:** Department of Biological Sciences, University of Manitoba, Winnipeg, Manitoba, Canada

**Keywords:** Subfornical organ, Patch clamp electrophysiology, Sensory circumventricular organ, Energy homoeostasis, Cardiovascular output

## Abstract

The subfornical organ (SFO) is a sensory circumventricular organ, lacking a blood-brain barrier. It is well-recognized as a key center for detection and integration of osmotic, ionic and hormonal signals for maintenance of hydromineral balance and cardiovascular regulation. Recently, the SFO has also been recognized as a center for the detection and integration of circulating satiety signals for regulation of energy balance. Neuropeptide Y (NPY) is a multifunctional neuropeptide, with effects on energy balance, cardiovascular tone and other aspects of homeostasis. Interestingly, despite the overlap of function between SFO and NPY, and observations that SFO expresses several subtypes of Y receptors, NPY regulation of SFO neurons has never been investigated. In this study, we examined the effects of NPY on dissociated rat SFO neurons using patch clamp electrophysiology. We observed that 300 nM NPY caused depolarization of 16 % of SFO neurons tested, and hyperpolarization of 26 %, while the remaining neurons were insensitive to NPY (n = 31). These effects were dose-dependent with an apparent EC_50_ of 3.9 nM for depolarizing neurons and 3.5 nM for hyperpolarizing neurons. Activation of Y5 receptors alone led to predominately hyperpolarizing effects, while activation of Y1 or Y2 receptors alone led to mixed responses. Voltage-clamp experiments demonstrated that NPY caused increases in voltage-gated K^+^ current amplitude as well as hyperpolarizing shifts in persistent Na^+^ current, mediating the hyperpolarizing and depolarizing effects, respectively. These findings indicate that NPY elicits direct electrophysiological effects on SFO neurons, suggesting that NPY acts via the SFO to regulate energy homeostatic function.

## Highlights

• The subfornical organ (SFO) of the brain lacks a blood brain barrier
• The SFO plays roles in regulation of hydromineral balance and cardiovascular tone
• SFO expresses Y1, Y2, Y5 isoforms of NPY receptors
• Application of NPY dose dependently affects excitability of SFO neurons
• Activation of only Y5 receptors results in only hyperpolarization on SFO neurons

## Graphical abstract

**Figure:**
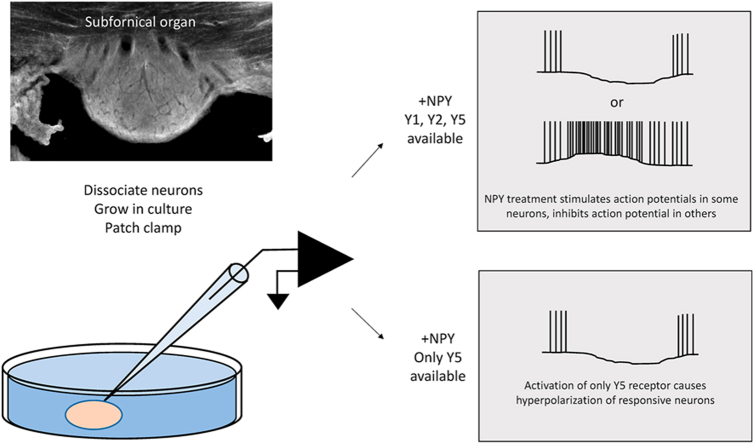

## 1 Introduction

Detection and integration of neurohumoral signals is critical to the regulation of homeostasis. The subfornical organ (SFO) is a sensory circumventricular organ (CVO), found on rostral wall of the third ventricle, that lacks a complete blood-brain barrier (Gross, 1991). Neurons of the SFO are endowed with a wide variety of receptors suited to detecting blood born signals such as hormones, satiety signals, metabolites, ionic concentration and osmolality (Hindmarch et al., 2008; Smith and Ferguson, 2010). Additionally, SFO neurons form synaptic connections to numerous autonomic control centers including the paraventricular nucleus of the hypothalamus (PVN), supraoptic nucleus of the hypothalamus (SON), arcuate nucleus of the hypothalamus, bed nucleus of the stria terminalis, medial preoptic nucleus and lateral hypothalamus (reviewed in (McKinley et al., 2003)). Thus SFO neurons are positioned to play important roles in detecting circulating signaling molecules, and communicating this information to higher control areas that regulate homeostatic parameters.

SFO is well-recognized for its key role in hydromineral and cardiovascular regulation, in part because of its ability to detect circulating angiotensin II (ANGII) and vasopressin (McKinley et al., 2003; Ferguson and Bains, 1997). A role for SFO in regulating thirst was first indicated by the experiments of Simpson and Routtenberg (1973), and also more recently demonstrated using optogenetic and DREADD technologies respectively (Nation et al., 2016; Oka et al., 2015). SFO neurons are also sensitive to varying concentrations of ions including Na^+^ (Hiyama et al., 2002) and Ca^++^(Quinn et al., 1998). In addition to hydromineral and cardiovascular regulation, SFO is also recognized for its emerging role in regulation of energy balance. For example, direct electrical stimulation of SFO elicits food intake (Smith et al., 2010). Lesion of SFO inhibits response to anorexigenic peptides (Baraboi et al., 2010). The SFO exhibits a wide variety and high density of receptors for satiety signals (Hindmarch et al., 2008), and electrical activity SFO neurons is directly modulated by many of these satiety signals including ghrelin, amylin, insulin, leptin, adiponectin and others (Smith and Ferguson, 2010; Pulman et al., 2006; Lakhi et al., 2013; Smith et al., 2009; Alim et al., 2010).

Neuropeptide Y (NPY) is a well-conserved 36 amino acid neuropeptide that belongs to the pancreatic peptide family. It is the most abundant neuropeptide in the brain, and binds to the family of G-protein coupled Y receptors. The Y receptors include Y1, Y2, Y4, Y5, and Y6. The Y1, Y2,and Y5 receptors are expressed throughout the CNS and periphery, but Y4 is mostly restricted to the periphery and has lower affinity for NPY (Zoccali et al., 2021). The Y6 receptor is non-functional in rats and primates. The Y receptors typically couple to Gi/Go family of proteins (Hirsch and Zukowska, 2012). NPY preferentially binds Y1, Y2 and Y5 receptors. These receptors are located throughout the CNS and periphery, implicating NPY in numerous physiological responses including hydromineral balance (Edvinsson et al., 1983; Pau et al., 1988), stress (Heilig, 2004), immune function (Wheway et al., 2005), circadian rhythms (Wiater et al., 2011) and autonomic responses (Kishi et al., 2005), however; NPY’s role in cardiovascular output (Zukowska-Grojec et al., 1988, Gruber et al., 2009) and energy regulation (Stanley et al., 1984; Elmquist et al., 1999; Yang et al., 2009) are the predominant effects of interest.

NPY is expressed in several central areas, however NPY-expressing neurons of the arcuate nucleus of the hypothalamus are key neurons in the regulation of energy balance. Major targets for NPY-ergic fibers such as the PVN, have roles in regulation of feeding and hydromineral and cardiovascular regulation (Elmquist et al., 1999). Intracerebroventricular NPY increases sympathetic drive and inhibition of the baroreceptor reflex (Martin et al., 1989; Vallejo and Lightman, 1986). NPY is also released into the circulation by chromaffin cells of the adrenal medulla due to stress (Hirsch and Zukowska, 2012; Cavada et al., 2002), by immune cells (Bedoui et al., 2003) or by the sympathetic nerve terminals of the gut and pancreas (Sundler et al., 1983). NPY in the periphery elicits robust pressor effects via Y1 and Y2 (Modin et al., 1991). Gruber et al. (Gruber et al., 2009) demonstrated that effects of peripheral NPY on cardiovascular regulation were rapid, suggesting an action via sensory circumventricular organs.

It is evident that the SFO and NPY each play key roles in energy balance and cardiovascular regulation. Given the strong association of obesity and cardiovascular disease, there have been suggestions of a link or common factor that could explain how pathological central dysregulation of energy homeostasis could affect cardiovascular regulation (Gruber et al., 2009). For example, it has been demonstrated through microarray experiments, in situ hybridization, and immunohistochemistry that Y1, Y2 and Y5 receptors are expressed in the SFO; the Y4 receptor is absent (Hindmarch et al., 2008; Kishi et al., 2005; Peterson et al., 2018; Dumont et al., 2000). Moreover, extensive immunostaining of NPY-containing fibers have been observed in rat and tree shrew SFO (Ni et al., 2015). However, the electrophysiological effects of NPY on SFO neurons are unknown. Therefore, in this study, we investigated potential roles of NPY in modulating the electrical excitability of SFO neurons and thus contributing to the effects of this peptide in the regulation of feeding and/or cardiovascular regulation. Using the patch-clamp technique, we provide direct demonstration that NPY modulates electrical activity of SFO neurons, and provide support for the notion that energy balance and cardiovascular regulation may be linked via central mechanisms.

## 2 Materials and methods

*Cell culture.* All animal protocols conformed to the standards of the Canadian Council on Animal Care and the University of Manitoba Animal Care Committee. Dissociated cultures of SFO neurons were prepared as previously described (Lakhi et al., 2013; Fry and Ferguson, 2007). Briefly, for each set of cultures three male Sprague-Dawley rats (∼150 g/5–6 weeks old) were decapitated, the brains quickly removed and placed into oxygenated, ice-cold brain slicing solution for 2 min containing the following (in mM): 87 NaCl, 2.5 KCl, 1.25 NaH_2_PO_4_, 0.5 CaCl_2_, 7 MgCl_2_-6H_2_O, 25 NaHCO_3_, 25 d-glucose, and 75 sucrose. A 3 mm slice beginning at the optic chiasm (approximately 9 mm–6 mm in the coordinates of (Paxinos and Watson, 2007) was prepared and transferred into Hibernate A media (Gibco, Burlington, ON, Canada) supplemented with 1X B-27 (Gibco). SFO was carefully dissected from surrounding tissue, transferred to 5 mL Hibernate A media containing 10 mg of papain (Worthington Biochemical Corp., Lakewood, NJ), and incubated for 25 min at 30 °C. Tissue was washed in Hibernate media/B-27, triturated, centrifuged at 200 *g* for 5 min, and suspended in Neurobasal-A/B-27 supplemented with 0.25X Glutamax (Gibco). Dissociated neurons were then plated on glass-bottomed 35-mm culture dishes (MatTek Ashland, MA) at a low density to ensure synaptic contacts did not form between neurons. The neurons were incubated at 37 °C in 5 % CO_2_. All experiments were performed within 1–4 days (Ferguson et al., 1997). Only neurons that had no contact to other cells were used for recordings, and only one neuron per dish was used. Cultured SFO neurons were visualized under a Zeiss IM35 inverted microscope (Zeiss, Germany) equipped with 40X Hoffman Modulation contrast optics and subjected to patch clamp electrophysiology.

*Electrophysiology.* Whole cell patch clamp recordings from SFO neurons were made using a HEKA EPC10 patch clamp amplifier and PatchMaster v2X90 (HEKA instruments, Mahone Bay, NS). Only neurons with a whole cell seal resistance >1 GΩ were used for further recordings. In the current clamp configuration, data were filtered at 10 kHz and acquired at 40–50 kHz. Patch electrodes had a resistance of 6.5–7.5 MΩ when filled with internal recording solution containing (in mM): 130 K-gluconate, 10 KCl, 2 MgCl_2_-6H_2_O, 5.5 EGTA, 10 HEPES, 0.1 CaCl_2_, and 2 Na_2_ATP, 0.5NaGTP, pH 7.3 with KOH. The external recording solution contained the following (in mM): 140 NaCl, 2 KCl, 2 CaCl_2_, 1 MgCl_2_-6H_2_O, 10 HEPES salt, and 10 glucose, pH 7.4 with NaOH. Osmolarity of intracellular and extracellular solutions was 295–300 mOsm. For voltage-clamp experiments, data were filtered at 10 kHz and acquired at 20–50 kHz. Patch electrodes for voltage-clamp experiments had a resistance of 3–4 MΩ. Series resistance was less than 30 MΩ. Voltage-clamp experiments were carried out using the same internal and external recording solutions. Junction potentials were eliminated using KCl pipette and ground electrodes (Shao and Feldman, 2007).

Chemicals for solutions were purchased from Sigma (Oakville, Ontario, Canada). NPY and antagonists (BIBO 3304, BIIE 0246, and L-152,804) were purchased from Tocris Biosciences (Bristol, UK). The maximum dose of NPY (300 nM) was chosen as to elicit a maimum electrophysiological effect based on previously published patch clamp studies (Gruber et al., 2009; Seki et al., 2020) where maximum effect was observed between 100 nm and 1 μM NPY; normal, unstressed concentration in rat plasma is near 50 pM, indicating 300 nM would be significantly elevated (Ding et al., 2024).

A solenoid-controlled perfusion system was used to apply NPY and antagonists to neurons in culture. This system allowed for exchange of external recording solution with the treatment, as well as efficient wash out. A neuron was considered responsive if a hyperpolarization or depolarization of more than 5 mV was observed within 200s: 5 mV was measured to be at least twice the standard deviation of resting membrane potential (Pulman et al., 2006; Smith et al., 2009; Alim et al., 2010).

*Data analysis.* Response of SFO neurons to 300 nM to 1 pM NPY was determined by comparing the membrane potential of neurons before and after application. Dose response curves were fitted with the Hill equation to determine the EC_50_. Changes in firing frequency and input resistance were determined before and after NPY application was measured.

Persistent Na^+^ currents (I_NaP_) were investigated using a depolarizing ramp protocol: the cell was held at −60 mV, hyperpolarzed to −80 mV and ramp depolarized to 0 mV over 80 ms. Amplitude of the peak I_NaP_ and the membrane potential at which the peak I_NaP_ current occurred were determined before and after application of 300 nM NPY. Outward voltage-gated K^+^ currents were elicited by a series of depolarizations. The peak amplitude of the rapidly inactivating K^+^ current was determined by measuring the outward current during the first 20 ms of membrane depolarization, and the delayed rectifier K^+^ current was determined by measuring the mean current from 90 to 100 ms during a 100 ms depolarization. Amplitude of the rapidly inactivating and delayed rectifier K^+^ currents were converted to density by dividing the current amplitude by the cell capacitance. The density-voltage relationships were determined before application of 300 nM NPY, and compared to values after application.

Continuous current clamp recordings were analyzed in Spike 2 Software (Version 6.18, Cambridge Electronic Design, Cambridge, UK). All statistical analyses were performed using OriginPro software (Northampton, MA, USA). Level of significance was considered to be p ≤ 0.05.

## 3 Results

*NPY modulates electrical properties of SFO neurons.* In the first series of experiments, current-clamp recordings from SFO neurons were obtained and the effect of NPY was examined. Recordings from 132 neurons were obtained with a mean resting potential of −60.4 ± 0.6 mV. Of the 132 neurons observed, 40.1 % were spontaneously active, with a firing frequency of 2.7 ± 0.4 Hz. The mean input resistance of all the neurons recorded was 4.4 ± 0.3 GΩ, similar to that reported for other studies.

**Fig. 1:**
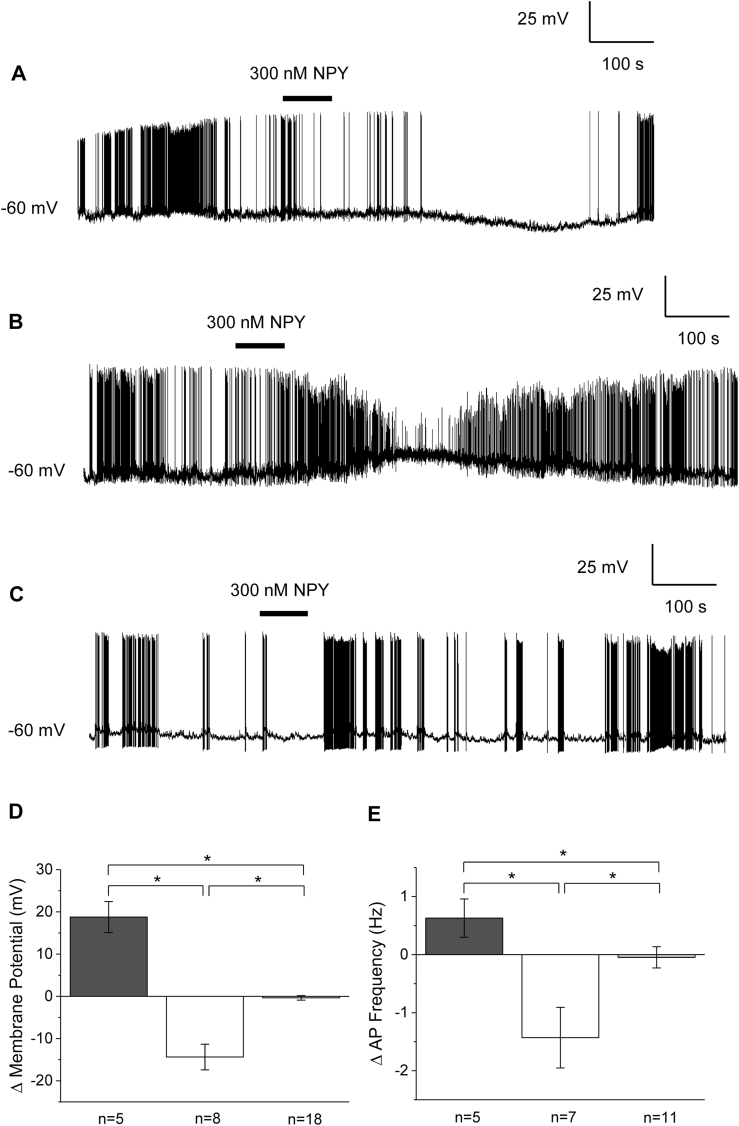
NPY modulates electrical activity of SFO neurons. Representative current-clamp recordings from neurons that were depolarized (A), hyperpolarized (B), or insensitive to applied NPY (C). Bar graphs representing mean changes in membrane potential (D) and action potential frequency (E) for cells treated with 300 nM NPY. Dark grey bars denote hyperpolarized; open bars denote hyperpolarized, light grey denote insensitive. The mean changes in membrane potential and action potential frequency were significantly different between all groups using the Kruskal-Wallis nonparametric ANOVA followed by Dunn’s multiple comparison test. Neurons that were quiescent before hyperpolarization were not included in the analysis. ∗P < 0.05.

The sensitivity of neurons to NPY of varying concentrations was tested, beginning with 300 nM, a concentration expected to elicit a near maximum response (Brown et al., 1995; McCullough et al., 1998). At this concentration, 16 % (5/31) of the neurons tested exhibited a depolarization in membrane potential (18.8 ± 3.7 mV), 26 % (8/31) exhibited a hyperpolarization in membrane potential (−14.4 ± 3 mV), and 58 % (18/31) exhibited no change, according to the criteria established in the methods sections (Fig. 1A–C). These effects were reversible in most neurons tested. Using the Kruskal-Wallis non-parametric ANOVA, changes in membrane potential were considered significantly different (p ≤ 0.05; Fig. 1D).

In addition to a change in membrane potential, application of 300 nM NPY caused a concomitant change in action potential frequency. In many neurons that depolarized, we observed an increase in action potential frequency by 0.6 ± 0.3 Hz and similarly, many neurons that hyperpolarized exhibited a decrease in frequency of 1.4 ± 0.5. Neurons that had no change in membrane potential did not demonstrated a significant change in action potential frequency (change of 0.0 ± 0.2 Hz). Using the Kruskal-Wallis non-parametric ANOVA, changes in action potential frequency were considered statistically significant between all groups (p ≤ 0.05; Fig. 1E).

**Fig. 2:**
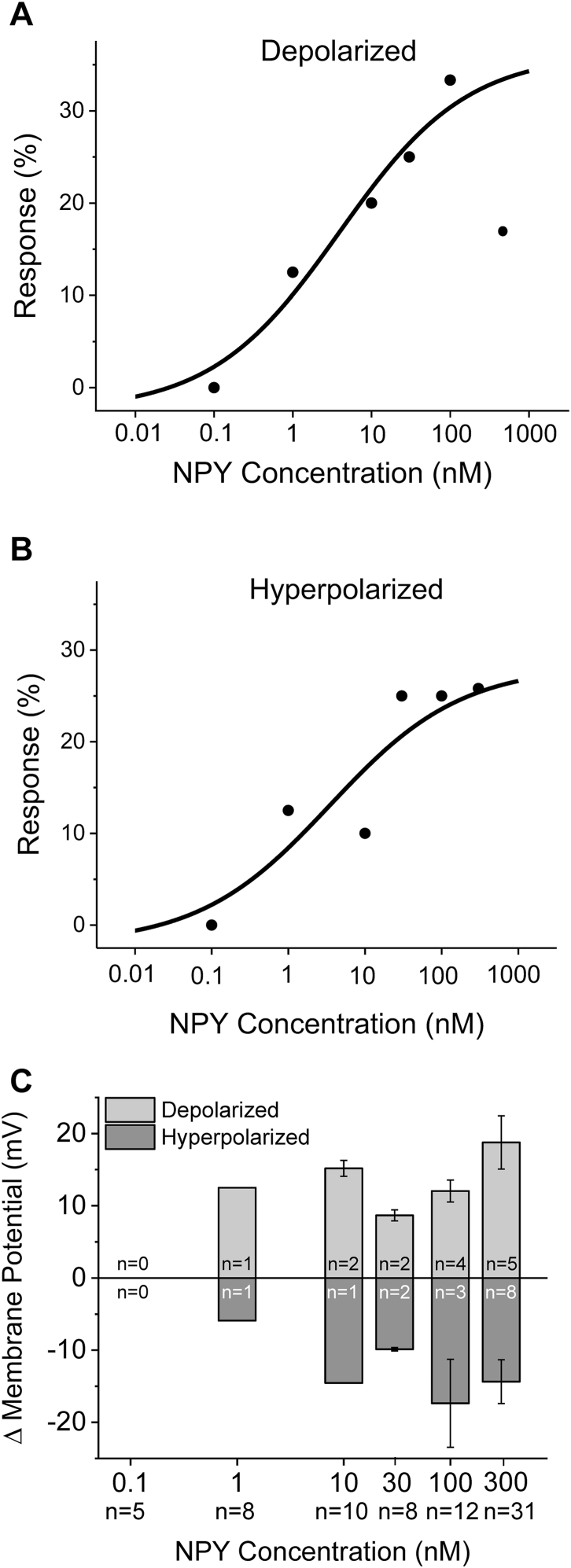
Effects of NPY are concentration dependent. *A,B*: percentage of neurons that responded to application of NPY increased with increasing concentrations. The data were fitted with the Hill equation, revealing an apparent EC50 of EC_50_ of 3.9 ± 2.9 nM for neurons that depolarized (A) and an EC_50_ of 3.5 ± 5.7 nM for neurons that hyperpolarized (B). *C*: change in membrane potential elicited by increasing concentration of NPY.

In order to determine whether the effects were concentration dependent, a dose-response was conducted using concentrations from 300 nM to 1 pM NPY. As illustrated in Fig. 2A and B as the concentration of NPY decreased, the percentage of neurons that depolarized or hyperpolarized in response to NPY decreased. The data were fitted to the Hill equation, revealing an EC_50_ of 3.9 ± 2.9 nM for neurons that depolarized and an EC_50_ of 3.5 ± 5.7 nM for neurons that hyperpolarized. Changes in membrane potential are shown in Fig. 2C.

**Fig. 3:**
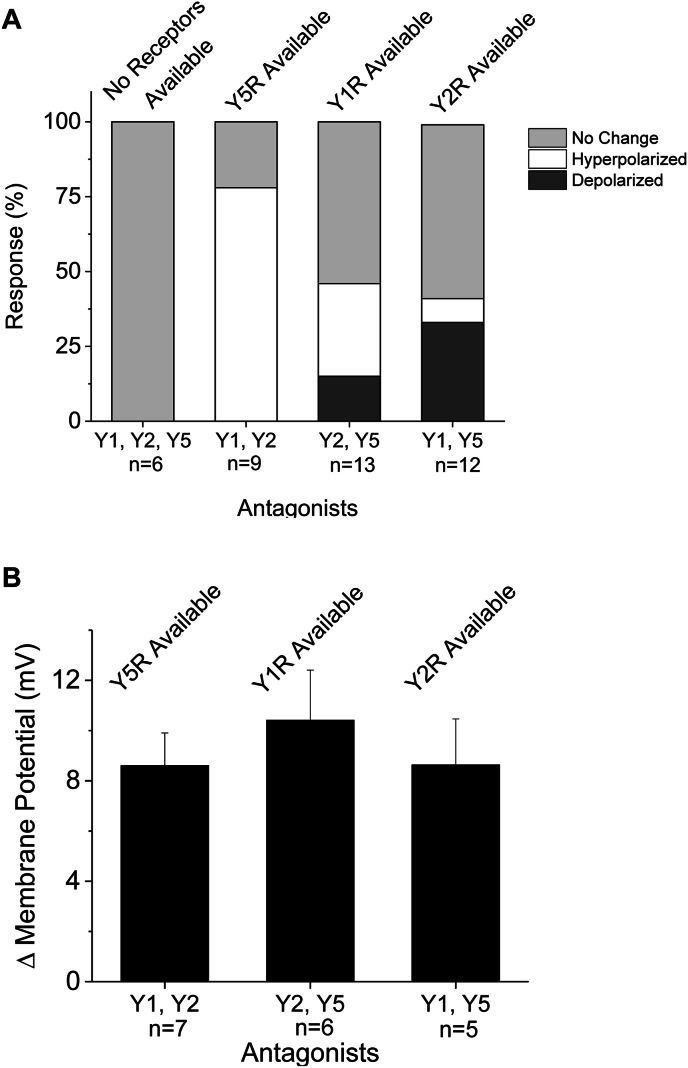
NPY stimulates multiple Y receptors in SFO neurons. *A:* A stacked bar graph representing the percent response of dissociated SFO neurons categorized by no change, a depolarization, or a hyperpolarization in membrane potential in response to the application of 100 nM NPY and specific Y receptor antagonists. Antagonists were applied at least 10 min prior to NPY application (100 nM Y1R antagonist BIB0 3304; 100 nM Y2R antagonist BIIE 0246; 1 μM Y5R antagonist L-152,804). The numbers below bars represent number of neurons tested each antagonist combination. *B:* Absolute change in membrane potential observed in responding neurons. Number below bars represent number of neurons responding to NPY in the presence of antagonist combination.

*NPY activates multiple Y receptors.* Rat CNS expresses five Y receptors, of which Y1, Y2 and Y5 are expressed in the SFO. In order to determine if the observed depolarizations and hyperpolarizations were caused by specific receptor subtypes, we carried out a series of current-clamp recordings of SFO neurons, applying NPY in the presence of specific Y receptor antagonists. In these experiments two of the three receptors were blocked at a time, leaving one subtype available, to determine which Y receptors were activated by NPY. In the presence of all three Y receptor antagonists (Y1R antagonist BIBO 3304; Y2R antagonist BIIE 0246; Y5R antagonist L-152,804), none of the neurons tested (0/6) exhibited a change in membrane potential, significantly different than the response to NPY alone (p = 0.0489; Chi-squared, 1DF). In the presence of the Y1R and the Y2R antagonists, allowing only activation of the Y5 receptor, we observed 7/9 of neurons tested hyperpolarized and 2/9 showed no change in membrane potential (Fig. 3A). In the presence of the Y2R and the Y5R antagonists, allowing only activation of the Y1 receptor, 2/13 neurons tested depolarized, 4 hyperpolarized, and 7/13 exhibited no change in membrane potential (Fig. 3A). Lastly, in the presence of the Y1R and Y5R antagonists, allowing only activation of Y2, we observed 4/12 neurons tested depolarized, 1/12 hyperpolarized, and 7/12 exhibited no change in membrane potential (Fig. 3A). The corresponding absolute value of the changes in membrane potential were 8.6 ± 1.3 mV, 10.4 ± 2.0 mV and 8.6 ± 1.8 mV respectively (Fig. 3B).

*NPY modulates multiple ion currents.* Current-clamp experiments using hyperpolarizing current injections revealed that I_Sag_ (a current carried by I_h_/HCN channels) was present in 43 % of neurons, however it was not modulated by NPY (data not shown). In order to further explore what ion currents are modulated by NPY, leading to depolarization or hyperpolarization of membrane potential, we carried out a series of voltage-clamp experiments. In these experiments ion currents of SFO neurons were recorded before and after application of 300 nM NPY.

**Fig. 4:**
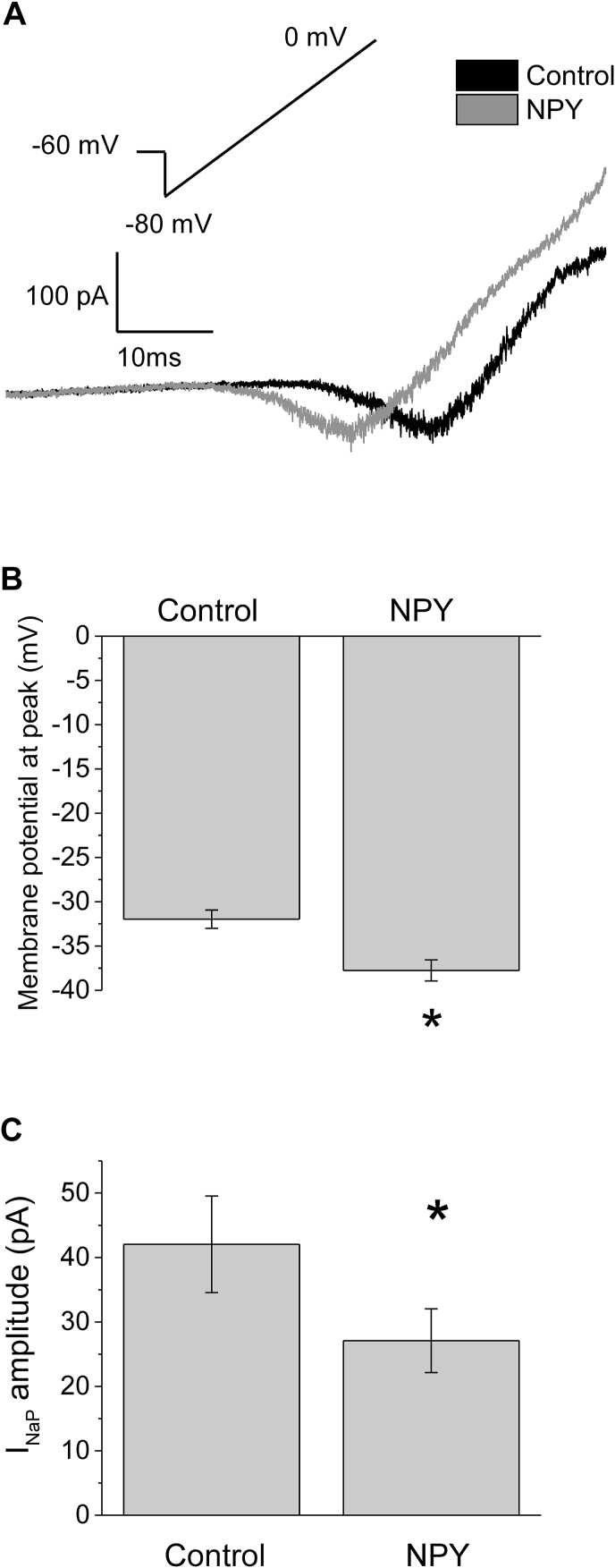
NPY modulates I_NaP_ in SFO neurons. *A:* A representative voltage clamp trace of an SFO neuron subjected to a depolarizing voltage ramp −80 to 0 mV before and after 300 nM NPY was applied. This neuron exhibited a 13 mV hyperpolarizing shift in the membrane potential at peak. *B:* Mean membrane potential at which the I_NaP_ peaked was significantly hyperpolarized by application of 300 nM NPY (paired *t*-test, n = 20, p ≤ 0.05, n = 20). *C:* Mean I_NaP_ amplitude was decreased by application of 300 nM NPY (paired *t*-test p ≤ 0.05, n = 16).

Modulation of I_NaP_ and NSCC have previously been demonstrated to alter electrical activity of SFO neurons in response to receptor activation (Pulman et al., 2006; Washburn et al., 2000). Therefore we investigated whether or not these currents were modulated by NPY. Neurons were subjected to depolarizing ramps in the absence and presence of 300 nM NPY (Fig. 4A). There was no evidence of modulation of NSCC (not shown), however there was evidence of modulation of I_NaP_. Analysis of these data revealed a significant hyperpolarizing shift in the membrane potential at which the peak current occurred in many but not all neurons, suggesting modulation of the voltage dependence of activation (Fig. 4A and B). Specifically, the mean peak membrane potential at which peak occurred was −31.9 ± 1.0 mV in control, but shifted to −37.8 ± 1.2 mV after NPY treatment (p < 0.05, n = 16, paired *t*-test; Fig. 4B). This shift in the voltage dependence of activation of I_NaP_ would tend to increase electrical activity of neurons. Paradoxically however, there was also a decrease in I_NaP_ amplitude after 300 nM NPY application in many, but not all neurons: specifically the I_NaP_ amplitude changed from a mean of 42.1 ± 7.5 pA, to 27.1 ± 4.9 pA (p ≤ 0.05, n = 16, paired *t*-test; Fig. 4C). Such a shift would tend to inhibit action potential activity in those neurons where it occurred. Interestingly, there was no significant correlation between the change in I_NaP_ amplitude and membrane potential where peak occurred (r^2^ = 0.07), reflecting the fact that in some neurons, there was little to no change in I_NaP_, but there was still a hyperpolarizing shift in its activation, and conversely, in some neurons with a relatively large decrease in I_NaP_, with little to no shift in activation (data not shown). Changes in amplitude and membrane potential at peak were not observed when extracellular recording solution was applied (paired *t*-test, n = 7, p = 0.74).

**Fig. 5:**
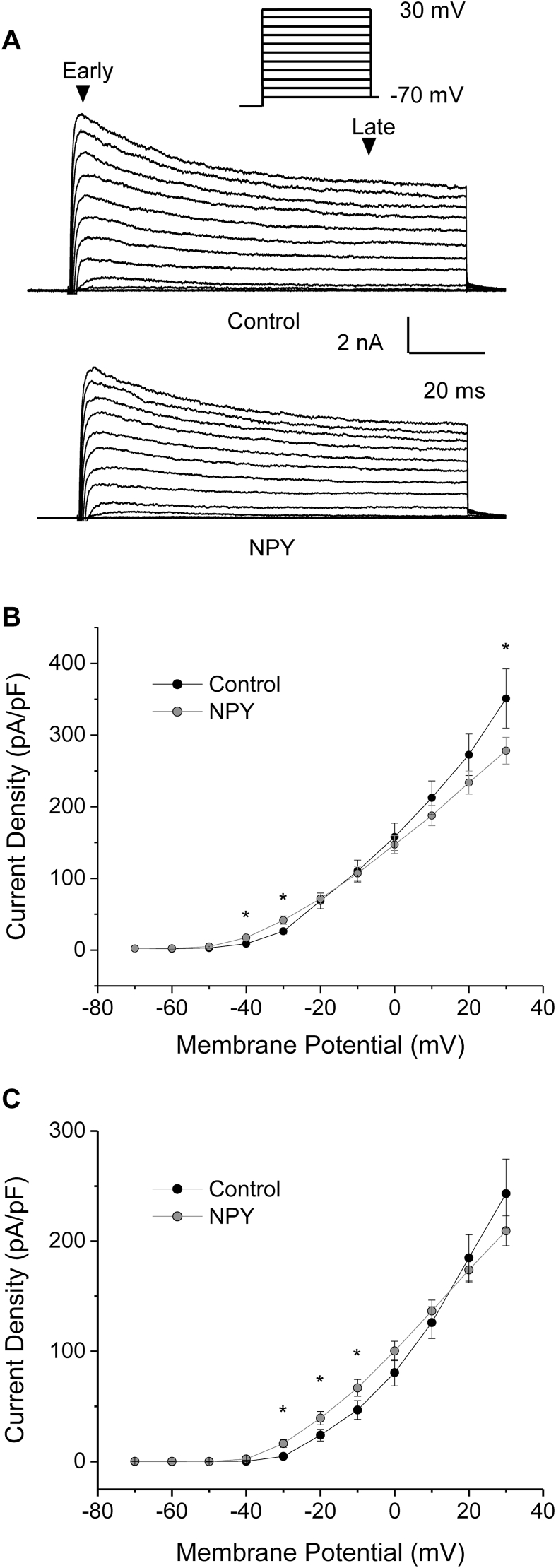
NPY modulates voltage-gated K^+^ currents in SFO neurons. *A:* Representative voltage clamp recordings of SFO neurons subjected to steps of increasing voltage from −70 to +30 mV for 100 ms before and after application of 300 nM NPY. *B:* Current density plots demonstrating at the early time point, NPY caused a significant increase in the voltage dependent activation of K^+^ current at −40 and −30 mV. *D:* At the late time point NPY also caused a significant increase in voltage-gated K^+^ current at −30, −20 and −10 mV (paired t-tests, p < 0.05, n = 16).

Neurons were also subjected to a series of depolarizing voltage steps before and after application of 300 nM NPY to determine whether or not modulated the voltage-gated K^+^ current (Fig. 5A). Voltage-gated K^+^ current density was analyzed at early and late time points to observe changes in the rapidly inactivating and delayed rectifier voltage-gated K^+^ currents, respectively. There was a significant increase in the rapidly inactivating K^+^ current density at −40 and −30 mV, and a significant decrease at +30 mV (paired *t*-test, p ≤ 0.05, n = 16; Fig. 5B). During the late time point, there was a significant increase in the delayed rectifier voltage-gated K^+^ current density at −30, −20, and −10 mV (paired t-tests, p ≤ 0.05, n = 16), demonstrating a leftward shift of voltage-gated K^+^ current activation due to NPY (Fig. 5B).

## 4 Discussion

Previous studies have confirmed the expression of Y1, Y2 and Y5 receptors in the SFO (Hindmarch et al., 2008; Kishi et al., 2005; Peterson et al., 2018; Dumont et al., 2000), however effects of NPY on electrical activity of SFO neurons have not been investigated. The present study used patch clamp electrophysiology to demonstrate that acutely applied NPY elicits dose dependent effects on the electrophysiological properties of SFO neurons. In contrast to previously published reports where NPY elicited only hyperpolarizing effects in responsive neurons for example refs (Melnick, 2012; Giesbrecht et al., 2012), we report here that acutely dissociated SFO neurons treated with NPY may exhibit hyperpolarizations or depolarization (or no response). Voltage clamp experiments showed that NPY caused modulation of two ion currents, persistent Na^+^ current and voltage gated K^+^ current, providing a mechanism for depolarization or hyperpolarization. The present study also used selective Y receptor antagonists, blocking two receptors at a time to reveal the effects of stimulating the unblocked Y receptor. The data suggest that all three Y receptors expressed in the SFO may be stimulated by NPY. Interestingly, stimulation of Y5 (in the presence of Y1 and Y2 antagonists) resulted in a predominantly hyperpolarization response, whereas stimulation of either Y1 or Y2 caused either depolarization or hyperpolarization. While the extent of overlap of the three receptor subtypes is not known, the fact that stimulation of Y5 in the presence of Y1 and Y2 blockade resulted in a higher proportion of NPY responding (with hyperpolarization) neurons than under conditions with no NPY and no antagonists suggests two key points: firstly, that Y1 and/or Y2 are co-expressed with Y5, and secondly that activation of Y1 and/or Y2 contributes to depolarization in those neurons inhibited by Y5 signaling.

What is the role of NPY acting at SFO? The SFO lacks a blood-brain-barrier, and is an important central site for detecting circulating signals critical for the regulation of numerous homeostatic functions including immune response, hydromineral balance, sympathetic output and energy balance, among others (Smith and Ferguson, 2010; McKinley et al., 2003). For example, SFO detects and responds to inflammatory signals including lipopolysaccharide (Xia and Krukoff, 2001; Takahashi et al., 1997) as well as circulating cytokines including interleukin-1 beta (IL-1β) (Wei et al., 2018), tumour necrosis factor alpha (Simpson and Ferguson, 2018), and interleukin-6 (Harré et al., 2003). The detection of circulating ANGII at the SFO has long been recognized as a primary stimulator of thirst (Simpson and Routtenberg, 1973; Ferguson et al., 1984), and ANGII acting at the SFO also increases sympathetic output and arterial blood pressure via stimulation of preautonomic neurons in the PVN (Ferguson et al., 1984; Ciriello and Gutman, 1991). Activation of SFO by ANGII also activates neurohypophysis neurons to regulate secretion of oxytocin and vasopressin (Li and Ferguson, 1993a, 1993b). SFO neurons have long been known to regulate Na^+^ appetite, and meal-associated drinking (Thunhorst et al., 1999); more recent studies by (Zimmerman et al., 2016) support the notion that food consumption and drinking are closely related ingestive behaviors because SFO neurons which promote thirst are also modulated by food ingestion (Zimmerman et al., 2016). While roles of the SFO in regulation of sympathetic output, thirst and secretion of neurohypophysial hormones are well-established, roles in energy balance are only beginning to be recognized. For example, in humans, lesion of SFO by autoimmune targeting of Na_X_ channel causes rapid onset obesity, together with other symptoms of autonomic dysregulation rapid weight gain (Ize-Ludlow et al., 2007). Several electrophysiological studies demonstrate that well-known energy balance signals such as insulin (Lakhi et al., 2013) and leptin (Smith et al., 2009) acutely cause excitation of some SFO neurons, and inhibition of others. Interestingly however, the action of some of these classic “energy balance” signals at the SFO also modulates autonomic function suggesting SFO to be an integrative metabolic center. For example, recently Jeong et al. (Kwon Jeong et al., 2019) demonstrated that loss of insulin signaling at the SFO (1) chronically lowers mean arterial pressure, and (2) causes significant weight gain and adiposity (Kwon Jeong et al., 2019). The neural pathways by which this happens remain to be elucidated, however they hypothesize the excitatory/inhibitory effect of insulin on SFO neurons (Lakhi et al., 2013) may relate to divergent cardio-metabolic effects. Current clamp experiments show leptin increased action potential firing of about 40 % of rat SFO neurons and hyperpolarized 25 % (Smith et al., 2009); when acutely injected into non-obese rats SFO, leptin decreased mean arterial pressure but the effect was lost in diet induced obese rats (Smith and Ferguson, 2012). In the present study, we identify that a subset of SFO neurons is depolarized by NPY, and another subset is hyperpolarized. Gruber et al. (Gruber et al., 2009), suggest that a role of circulating NPY acting at the SFO includes stimulation of pressor effects, and this hypothesis is supported by our present study. However it remains to be determined if circulating NPY acting at SFO also has a metabolic role. A recent study indicated that antagonism of peripheral Y1 receptors in mice by non-brain penetrating BIBO3304 prevented diet induced obesity by stimulating energy expenditure and thermogenesis (Yan et al., 2021). This effect is mediated by the action of BIBO3304 on adipose tissue, however a contribution by antagonism of Y1 in other peripheral sites including SFO could also contribute to these metabolic effects. Regarding NPY from afferent input to the SFO, the source of NPY-containing fibres is presently unknown (Ni et al., 2015), but arcuate nucleus, lateral hypothalamus and bed nucleus of the stria terminalis could be candidates (McKinley et al., 2003).

The antagonist studies indicate that activation of Y5 receptors predominantly causes a hyperpolarization effect, and that activation of Y1 and/or Y2 at least in part attenuates this response, or drives depolarization. While the reported electropgysiological effect of NPY on most neurons is inhibitory or hyperpolarizing (Melnick, 2012; Giesbrecht et al., 2012), several examples of mixed excitatory and inhibitory effects exist: in hamster submandibular ganglion neurons, NPY stimulated L-type Ca^++^ current via Gs in some cells, while inhibiting N- and P/Q type Ca^++^ currents via Gi in others (Endoh et al., 2012). Interestingly, one study reports activation of Y1 in lateral habenula may stimulate phospholipase C, not modulate the classic adenylyl cyclase pathway via Gi/Go (Cheon et al., 2020). Our voltage clamp experiment revealed that NPY modulated I_NaP_, and voltage gated K^+^ current. Both of these currents critically determine the overall excitability of SFO neurons. Washburn et al. (2000), first described the role of I_NaP_ in driving spontaneous activity and bursting activity in SFO neurons. More recently, Fry and Ferguson established that I_NaP_, within a narrow range on membrane potentials, drove subthreshold oscillations which lead to spontaneous activity and bursting (Fry and Ferguson, 2007). Therefore modulation of I_NaP_ can effectively and dramatically alter the electrical properties of SFO neurons. Of particular note, the changes in I_NaP_ amplitude and voltage of activation occurred independently of each other: there was no correlation between the change in amplitude of I_NaP_ and shift in membrane potential of the peak amplitude, indicating that for some neurons NPY modulation of I_NaP_ would act to facilitate action potential activity, but in other neurons, would tend to favor inhibition of action potentials. The mechanism underlying this variability is unclear, however it may result from differential modulation of voltage gated Na^+^ channel isoforms, Nav1.1,Nav1.2, Nav1.3, Nav1.6 and Nav1.7 in SFO neurons. (Hindmarch et al., 2008; Peterson et al., 2018). Importantly, Seki et al. reported NPY, acting on Y1 and Y5 depolarized rat mesencephalic V neurons via increasing amplitude of I_NaP_ and shifting voltage dependence of activation (Seki et al., 2020). Voltage gated K^+^ currents were also modulated by NPY, resulting in increased activity in the subthreshold range of −30 to −40 mV, suggesting that NPY may inhibit action potential generation via increasing K^+^ channel opening near resting membrane. Previous studies have also observed modulation of electrical activity in SFO neurons via K^+^ currents where vasopressin or ghrelin inhibited voltage gated K^+^ currents (Washburn et al., 1999) and nesfatin-1enhanced K^+^ currents (Kuksis and Ferguson, 2014). Taken together, our present voltage clamp data indicate that NPY can modulate I_NaP_ to stimulate or inhibit electrical activity, and voltage gated K^+^ currents to inhibit activity. Given that SFO neurons typically have high input resistances in the range of 1–5 GOhm, the net effect on action potential activity would depend on the balance of altering I_NaP_ and K^+^ current conductances near resting membrane potential.

Whether activation of either Y1, Y2, or Y5 in the SFO underlie specific might functions is unknown, but localization of receptor subtypes to specific regions of SFO may help reveal this. For example, in rat the SFO can be anatomically divided into rostocaudal core (cSFO) and shell (sSFO) subregions. Neurons of the cSFO project to rostral bed nucleus of the stria terminalis, organum vasculosum of the lamina terminalis, and the parvocellular subdivision of the PVN (Kawano and Masuko, 2010; Saper and Levisohn, 1983; McKinley et al., 1998). Conversely, sSFO neurons project to median preoptic nucleus and magnocellular neurons of SON and PVN (Kawano and Masuko, 2010; Jhamandas et al., 1989). The neurons projecting from SFO to the PVN are excitatory, not inhibitory (Ferguson et al., 1984; Bains and Ferguson, 1995), and also appear to use ANGII as neurotransmitter (Lind et al., 1985; Ferguson, 2009). In rats, the cSFO neurons can be identified by expression of calbindin, and sSFO by expression of calretinin (Huang et al., 2019), but otherwise there is no electrophysiological or morphological “fingerprint” to predict whether a neuron originated from the core or shell regions.

The notion that NPY elicits changes in the electrophysiological properties of SFO neurons is physiologically relevant as NPY plays a central role in both energy regulation and cardiovascular output and the SFO is a known integration site for both of these functions. Understanding the role of NPY at SFO is important then, especially in light of the co-occurrence of obesity and cardiovascular disturbances. Ni et al. (2015), have recently demonstrated the presence of NPY axons in the SFO, thus begging the questions of what is the biological purpose of NPY acting on SFO neurons, and is synaptic release or NPY from circulation the primary source of NPY acting at Y receptors in the SFO. The fact that also SFO receives neural input from NPY-expressing areas such as arcuate nucleus, lateral hypothalamus and bed nucleus of the stria terminalis (McKinley et al., 2003) suggests that NPY may regulate homeostatic function in as yet unappreciated ways.

## 5 Conclusion

The present study is the first to demonstrate that NPY modulates electrophysiological characteristics of SFO neurons. The observation that SFO neurons may be depolarized or hyperpolarized by NPY may be likely due to the simultaneous stimulation of multiple Y receptors Y1, Y2 and Y5. Activation of Y receptors modulates at least two key conductances, I_NaP_ and voltage gated K^+^ currents. These current changes are likely the key driving factors of the depolarizing and hyperpolarizing changes in membrane potential respectively. The current findings suggest that NPY acting on SFO may be a key process in the intersection of energy balance and cardiovascular regulation.

## CRediT authorship contribution statement

**Lauren Shute:** Conceptualization, Methodology, Formal analysis, Investigation, Writing – original draft, Writing – review & editing. **Mark Fry:** Conceptualization, Methodology, Resources, Data curation, Writing – review & editing, Visualization, Supervision, Project administration, Funding acquisition.

## Funding

This work was supported by a Natural Sciences and Engineering Council of Canada Discovery Grant.

## Declaration of competing interest

The authors declare that they have no known competing financial interests or personal relationships that could have appeared to influence the work reported in this paper.

## Data Availability

Data will be made available on request.
